# Nanosecond time-resolved infrared spectroscopy for the study of electron transfer in photosystem I

**DOI:** 10.1007/s11120-023-01035-9

**Published:** 2023-07-07

**Authors:** Sarah M. Mäusle, Neva Agarwala, Viktor G. Eichmann, Holger Dau, Dennis J. Nürnberg, Gary Hastings

**Affiliations:** 1 Department of Physics, Freie Universität Berlin, Arnimallee 14, 14195 Berlin, Germany; 2 Department of Physics and Astronomy, Georgia State University, Atlanta, GA 30303 USA; 3 Department of Chemistry, Georgia State University, Atlanta, GA 30303 USA; 4 Dahlem Centre of Plant Sciences, Freie Universität Berlin, Berlin, Germany

**Keywords:** Photosynthesis, Photosystem I, *Thermosynechococcus vestitus* BP-1, Nanosecond time-resolved infrared spectroscopy, Electron transfer, A_1_, P700

## Abstract

Microsecond time-resolved step-scan FTIR difference spectroscopy was used to study photosystem I (PSI) from *Thermosynechococcus vestitus* BP-1 (*T. vestitus,* formerly known as *T. elongatus*) at 77 K. In addition, photoaccumulated (P700^+^–P700) FTIR difference spectra were obtained at both 77 and 293 K. The FTIR difference spectra are presented here for the first time. To extend upon these FTIR studies nanosecond time-resolved infrared difference spectroscopy was also used to study PSI from *T. vestitus* at 296 K. Nanosecond infrared spectroscopy has never been used to study PSI samples at physiological temperatures, and here it is shown that such an approach has great value as it allows a direct probe of electron transfer down both branches in PSI. In PSI at 296 K, the infrared flash-induced absorption changes indicate electron transfer down the B- and A-branches is characterized by time constants of 33 and 364 ns, respectively, in good agreement with visible spectroscopy studies. These time constants are associated with forward electron transfer from A_1_^–^ to F_X_ on the B- and A-branches, respectively. At several infrared wavelengths flash-induced absorption changes at 296 K recover in tens to hundreds of milliseconds. The dominant decay phase is characterized by a lifetime of 128 ms. These millisecond changes are assigned to radical pair recombination reactions, with the changes being associated primarily with P700^+^ rereduction. This conclusion follows from the observation that the millisecond infrared spectrum is very similar to the photoaccumulated (P700^+^–P700) FTIR difference spectrum.

**Supplementary Information:**

The online version contains supplementary material available at 10.1007/s11120-023-01035-9.

## Introduction

In photosynthesis solar energy is harvested and used to synthesize chemical products that are ultimately the source of most of the food and fuels consumed by humanity today (Walker 1993). In oxygen evolving organisms two photosystems, called photosystem (PS) I and II, capture and convert solar energy independently, but cooperatively (Barber 1992; Walker 1993). The solar conversion reactions occur in a centralized pigment-protein unit called a reaction center. In the reaction center, light is used to drive electrons, via a series of acceptors, across a biological membrane (the thylakoid membrane). This light-induced separation of charge is the basic mechanism underlying solar energy capture in all photosynthetic organisms.

**Fig. 1:**
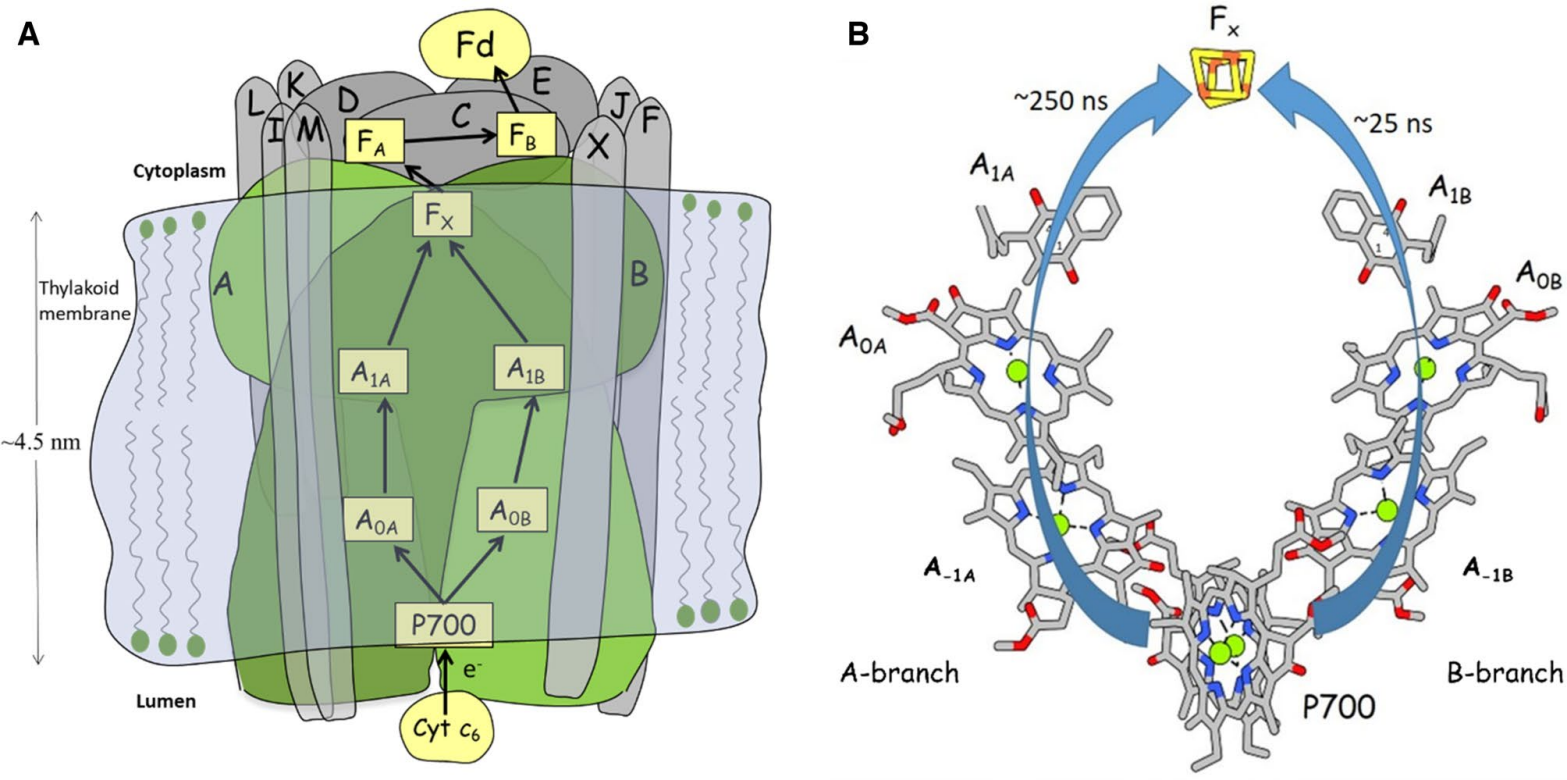
**A** Cartoon depicting the PSI complex embedded in the thylakoid membrane. ET paths between cofactors are indicated. Protein subunits are labeled A–X. **B** Arrangement of ET cofactors in PSI. Figure produced using the 2.5 Å crystal structure of PSI from *T. vestitus* (Jordan et al. 2001). Cofactor hydrocarbon tails have been truncated. Arrows indicate ET routes along with associated time constants. Cyt *c*_6_ is cytochrome *c*_6_, A_-1B_, A_-1A_, A_0B_ and A_0A_ are chlorophylls, A_1B_ and A_1A_ are phylloquinones, F_X_, F_A_ and F_B_ are iron-sulfur clusters and Fd is ferredoxin

In this manuscript we focus on electron transfer (ET) in isolated cyanobacterial PSI complexes from *Thermosynechococcus vestitus* BP-1 (previously known as *Thermosynechococcus elongatus* BP-1). PSI consists of 11–13 protein subunits, many of which have been characterized (Chitnis et al. 1995; Golbeck 1995; Pakrasi 1995). The ET cofactors are bound to the PsaA and PsaB membrane-spanning protein subunits (Fig. 1A) (Fromme and Grotjohann 2006). The terminal ET cofactors, F_A_ and F_B_, are bound to the stromal PsaC subunit (Fromme and Grotjohann 2006). The organization of the bound ET cofactors is outlined in Fig. 1B. The cofactor organization is near identical in PSI from plants, algae and cyanobacteria (Ben-Shem et al. 2003; Jolley et al. 2005; Malavath et al. 2018; Mazor et al. 2015, 2014; Qin et al. 2015a, b).

In isolated PSI particles, following light excitation, the P700^+^A_1_^–^ radical pair state is formed within ~ 50 picoseconds (Hastings et al. 1995, 1994). In PSI there are two cofactor branches with approximate C_2_ symmetry (Fig. 1B), referred to as the A– and B–branch. In this manuscript the A–branch refers to the side in which the pigment in the A_1_ binding site is bound to the PsaA protein subunit (A_1A_ in Fig. 1). ET can occur down both branches from P700 to F_x_ (Fig. 1B) (Makita and Hastings 2015; Redding and van der Est 2006).

Both the A_1A_ and A_1B_ pigments in PSI are phylloquinone (PhQ) molecules, in a very similar protein environment (Brettel 1997; Golbeck 1992; Golbeck and Bryant 1991; Srinivasan and Golbeck 2009). In cyanobacterial PSI at RT, forward ET from A_1_^–^ to F_X_ proceeds biphasically with time constants of ~ 25 and ~ 250 ns (Fig. 1B). The “fast” and “slow” time constants were observed in visible transient absorption spectroscopy experiments and are thought to be associated with ET from A_1B_^–^ and A_1A_^–^ to F_X_, respectively (Agalarov and Brettel 2003; Makita and Hastings 2015; Makita et al. 2015; Redding and van der Est 2006; Schlodder et al. 1998; van der Est 2006). At RT, ET from F_X_^–^ to F_A_ and then on to F_B_ occurs on a tens to hundreds of nanosecond timescale (Byrdin et al. 2006). In the absence of added electron acceptors, the P700^+^F_A/B_^–^ state recombines in 50–150 ms (Golbeck and Bryant 1991). The F_A/B_ terminology is used to indicate that we do not specifically distinguish between the F_A_ and F_B_ states.

In cyanobacterial PSI, forward ET from A_1_^–^ to F_X_ diminishes as the temperature is lowered, and is replaced by a P700^+^A_1A_^–^ recombination reaction, which is characterized by a time constant of ~ 300 microseconds at 77 K (Makita and Hastings 2015, 2016a, b; Makita et al. 2015; Schlodder et al. 1998). On the one hand, since ET recombination is essentially unidirectional down the A-branch at 77 K, spectral analysis is simplified. On the other hand, however, in microsecond time-resolved step-scan (TRSS) FTIR difference spectroscopy (DS) studies of PSI at 77 K, the bands associated with the P700^+^ and A_1_^–^ states decay with the same time constant, which negates the possibility of using the temporal evolution of bands to distinguish their origin. At room temperature (RT, 296 ± 2 K), P700^+^ and A_1_^–^ decay on different timescales, allowing the possibility of assigning spectroscopic signatures (bands) based on their temporal evolution, thus the time resolution itself allows an approach to infrared spectral band identification and assignment.

At RT, the P700^+^A_1B_^–^/P700^+^A_1A_^–^ states decay in ~ 25/250 ns, respectively, and can potentially be studied using TRSS FTIR DS. Nanosecond TRSS FTIR DS has been used to study a range of protein complexes (Lorenz-Fonfria 2020; Mezzetti 2020; Mezzetti et al. 2022). TR FTIR DS (using both step-scan FTIR and dispersive instruments) has been used to study photosynthetic proteins (Mezzetti and Leibl 2017), but no work has been done on photosynthetic proteins with time resolution near 50 ns (but see Mäusle et al. 2020). Nanosecond TRSS FTIR DS studies of large photosynthetic proteins such as PSI are difficult, as noise levels below 1 × 10^–5^ in OD units (0.01 mOD) are required (Hastings 2001, 2006). Such highly-sensitive ns-TRSS FTIR DS measurements are challenging (Hage et al. 1996; Hastings 2001, 2006; Rodig and Siebert 1999). Even microsecond-TRSS FTIR DS measurements with sensitivity near 0.01 mOD are challenging (Hastings 2006; Mezzetti 2020; Mezzetti and Leibl 2017). Recently, we have studied PSII using TRSS FTIR DS, and to get to the necessary sensitivity required multiple interchangeable samples and data acquisition over a period of several months (Greife et al. 2023). This would be considered unacceptable in most studies.

In summary, the main challenges in TRSS FTIR DS studies of PSI at RT, are the dual need for time resolution below 50 ns and sensitivity below 0.01 mOD. To the best of our knowledge these specifications have not been met using FTIR-based instrumentation, but are met using the new quantum cascade laser (QCL) based instrumentation outlined in this manuscript.

TRSS FTIR DS is nevertheless advantageous for collecting time-resolved data over a broad spectral region. The downside is that sensitivity is limited, as discussed above, especially on nanosecond timescales (Hastings 2001; Rammelsberg et al. 1997). An alternative approach is to undertake nanosecond time resolved infrared (TRIR) DS measurements at single frequencies using very intense IR light sources (with intense IR light sources more highly absorbing samples can be studied, which will yield more intense spectral signals, easing sensitivity requirements). QCLs can provide such high intensity IR light. Previously, QCLs were thought to be disadvantageous because of limited spectral coverage. However, we demonstrate here with our QCL setup that TR data can be collected with high spectral resolution covering the entire 1310–1890 cm^–1^ region. One advantage in investigation of PSI is that it is a highly robust and stable protein that can be subjected to literally thousands of laser flashes without appreciable degradation or damage. This allows us to undertake many separate single wavelength experiments covering the entire spectral region, with considerable signal averaging at each wavelength, allowing high signal to noise ratio at each of the wavelengths in the experiment.

In this manuscript we have used a QCL based pump-probe spectrometer to undertake the first nanosecond TRIR DS studies of PSI complexes in the physiological temperature range (~ 296 K). Such measurements have allowed us to probe absorption changes associated with single molecular bonds of the phylloquinone molecules on both the A– and B–branch as they undergo ET. To complement and aid in the analysis of the TRIR data, photoaccumulated (P700^+^–P700) FTIR DS were obtained at 77 and 293 K and (P700^+^A_1_^–^–P700A_1_) TRSS FTIR DS were obtained at 77 K. Time resolved and photoaccumulated FTIR DS for PSI from *T. vestitus* have not been presented previously.

## Materials and methods

Trimeric PSI particles from *T. vestitus* were prepared by first binding His-tagged PSII complexes to a nickel resin (Mäusle et al. 2020) and applying the PSI-enriched flow through to a Toyopearl DEAE-650S column as previously described (Kölsch et al. 2018). The eluted PSI fractions were concentrated using an Amicon Stirred Cell with a Synder LY membrane (Sterlitech, USA), frozen in liquid nitrogen and stored at − 80 °C.

Photoaccumulated (P700^+^ − P700) FTIR DS (at both RT and 77 K) and TRSS FTIR DS (at 77 K), all at 4 cm^–1^ spectral resolution, were collected as described previously (Agarwala et al. 2023; Makita and Hastings 2020). PSI samples for single-frequency TRIR DS experiments were prepared in a similar manner: After thawing on ice, the sample was diluted in 50 mM TRIS buffer (pH 8), and 0.04% β-DM, such that a chlorophyll concentration of 0.2 mg/ml was reached. The sample was centrifuged at 300,000 g for three hours at 4 °C. A CaF_2_ window was prepared with a ring of vacuum grease and a 15 µm spacer. Two artificial electron donors (0.1 µl of 20 µM PMS and 0.1 µl of 20 mM sodium ascorbate) were pipetted onto the CaF_2_ window, before adding a small amount of the soft PSI pellet and carefully mixing with a spatula. A second CaF_2_ window was placed onto the first, tightly sealing the sample in between.

TRIR DS measurements were performed using the previously described experimental setup (Mäusle et al. 2020) with some modifications: 1. The intensity of the pulsed 532 nm Nd:YAG laser is now regulated by a motorized attenuator (Eksma Optics, Vilnius, Lithuania). 2. The QCL system was replaced by a newer model (MIRcat-QT-Z-2300) with improved optical properties (wavenumber accuracy < 1 cm^–1^, covering the entire 1310–1890 cm^–1^ spectral range with improved pointing stability) (Daylight Solutions, San Diego, CA, USA).

For each desired wavelength, a flash sequence of two dark measurements (no flashes), followed by five saturating excitation flashes (~ 0.1 mJ/mm^2^) and a final flash with higher intensity (~ 0.3 mJ/mm^2^) was applied repeatedly to a single sample spot. The IR signal was measured from 20 ms before and up to 800 ms after each excitation flash. The difference in signal between the low and high excitation flashes was used to calculate a sample heating artefact, which was fit by a sum of exponentials and subtracted from the data (Fig. S1). Heating artefacts and their correction are well known in IR spectroscopy (Lorenz-Fonfria 2020; Mezzetti and Spezia 2008).

A pulse generator was used for triggering the excitation laser and data acquisition. The timing of the excitation flash was determined by recording each flash with a photodiode. The sample chamber was flushed with dry air and the temperature was set at 296 ± 2 K. We will refer to temperatures in this range as room temperature (RT).

At each frequency the results obtained for 600–6000 laser flashes were averaged. The data was then subjected to a global analysis procedure as follows: Transient IR absorption changes were fitted to a sum of exponential functions plus a constant [$y\left(t,\nu \right)= {y}_{o}+{\sum }_{i}{A}_{i,\upnu }{e}^{-t/{\tau }_{i}}$] using a least-squares approach implemented in Python 3.7 (Python Software Foundation, Delaware, USA). For the fits on the nanosecond timescale, the exponential function was convolved with an approximate instrument response function (a Gaussian with standard width of 17 ns) as illustrated in Fig. S2. For results obtained by an alternative approach, see Fig. S3 and S4. As is standard in these types of global analysis fitting procedures, the time constants were constrained to be the same at each wavelength, while the amplitudes (pre-exponential factors) were allowed to vary.

## Results

**Fig. 2:**
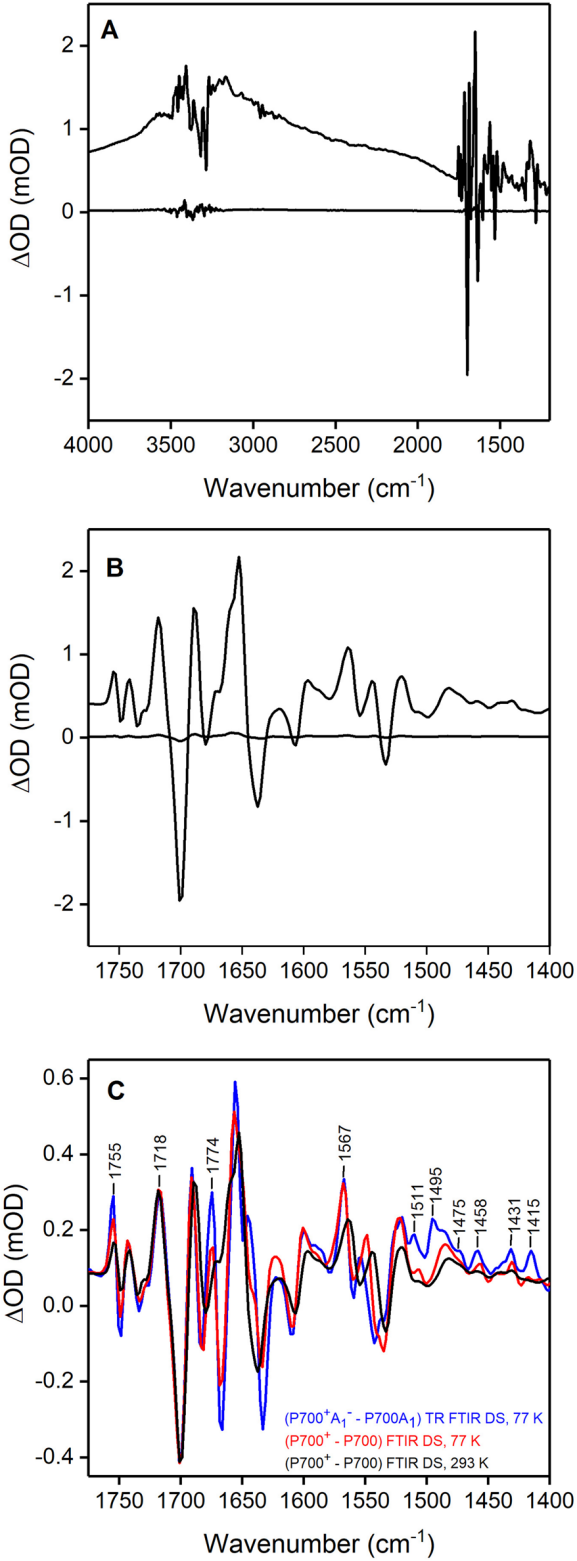
FTIR DS for PSI from *T. vestitus*: Photoaccumulated (P700^+^–P700) FTIR DS at RT in the **A** 4000–1200 and **B** 1775–1400 cm^–1^ regions. The “flat line” is a “dark–dark” FTIR DS that indicates the noise level in the experiment, as well as the zero-absorbance line. **C** Microsecond time-resolved (P700^+^A_1A_^–^–P700A_1A_) FTIR DS at 77 K (*blue*). This spectrum is the average of nine spectra collected in 6 μs increments following a laser flash. Photoaccumulated (P700^+^–P700) FTIR DS for PSI at 77 (*red*) and 293 K (*black*) are also shown in **C** (redrawn from **B**). Spectra were scaled to the 1718( +)/1700(–) cm^–1^ difference band. The absorbance scale in (**C**) is for the TR FTIR DS

Figure 2A shows photoaccumulated (P700^+^–P700) FTIR DS collected at RT for PSI from *T. vestitus* in the 4000–1200 cm^–1^ region. The dark minus dark spectrum indicates the noise level in the experiment as well as the zero-absorbance line. As is relatively well known (Breton 2001; Nabedryk et al. 2000) and is clearly seen in Fig. 2A, P700^+^ displays several very broad, positive, *electronic absorption bands* throughout the 4000–1200 cm^–1^ region. This broad positive electronic absorption difference overlies the IR difference bands in the 1800–1400 cm^–1^ region (Fig. 2B), essentially “pushing” the whole spectrum (of IR difference bands) more positive. Figure 2C shows microsecond TRSS (P700^+^A_1_^–^–P700A_1_) FTIR DS obtained using *T. vestitus* PSI samples at 77 K (*blue*). As has been described (Hastings 2015) this TRSS FTIR DS is the average of nine spectra, collected in consecutive 6 µs increments following a laser flash. This spectrum can therefore be considered as an averaged spectrum obtained over a ~ 54 µs time period. Photoaccumulated (P700^+^–P700) FTIR DS at both 77 (*red*) and 293 K (*black*) are also shown in Fig. 2C for comparison. Both the TRSS and photoaccumulated FTIR DS for PSI from *T. vestitus* are presented here for the first time.

Although we call the RT photoaccumulated FTIR DS in Fig. 2A (P700^+^–P700) FTIR DS, it is strictly speaking a (P700^+^F_A/B_^–^–P700F_A/B_) FTIR DS. The 77 K photoaccumulated FTIR DS is also strictly speaking a (P700^+^F_X_^–^–P700F_X_) FTIR DS. In the latter case this is because P700^+^F_A/B_^–^ formation, which occurs in ~ 35% of the PSI complexes, is irreversible at 77 K (Schlodder et al. 1998), and signals associated with P700^+^F_A/B_^–^ will not contribute in our experiments that involve repetitive illumination. However, P700^+^F_X_^–^ recombination can occur at 77 K, with a half lifetime of 5–100 ms, in ~ 20% of the PSI complexes (Schlodder et al. 1998). Under repetitive bouts of continuous illumination at 77 K the P700^+^F_X_^–^ state will be the dominant photoaccumulated species. P700^+^A_1_^–^ is unlikely to be photoaccumulated as it decays in ~ 300 µs at 77 K.

By adding a variety of electron donors and acceptors, and by studying PSI samples that lack either F_A/B_ (Breton et al. 1999; Hastings and Sivakumar 2001), or F_A/B_ and F_X_ (Hastings and Sivakumar 2001), it has been determined that the iron sulfur clusters do not contribute significantly in (P700^+^F_A/B_^–^–P700F_A/B_) FTIR DS in the 1800–1200 cm^–1^ region (Fe-S vibrations are expected below ~ 500 cm^–1^ (Chu et al. 2000; Chu et al. 1999), and this is the justification for calling these photoaccumulated spectra (P700^+^–P700) FTIR DS. Nonetheless one might expect electrochromic effects from protein or pigment species near the reduced iron sulfur clusters to contribute to some degree in photoaccumulated FTIR DS. In a more recent study of PSI site directed mutants we did suggest possible small contributions to photoaccumulated FTIR DS from modes that might be impacted by F_X_ reduction (Agarwala et al. 2020).

The 77 K (P700^+^A_1_^–^–P700A_1_) TRSS FTIR DS in Fig. 2C displays positive bands at 1567, 1511, 1495 and 1415 cm^–1^, which are absent in the 77 K photoaccumulated (P700^+^–P700) FTIR DS. These bands are therefore associated with A_1_^–^. The bands at 1495 and 1415 cm^–1^ are well known to be due to stretching vibrations of the C_1_$\dddot -$O and C_4_$\dddot -$O groups of the phyllosemiquinone anion in the A_1A_ binding site (Hastings 2015; Rohani et al. 2019). Given the similarity in the frequencies of the bands in the (P700^+^–P700) FTIR DS at 293 and 77 K (Fig. 2C), it is likely that the phyllosemiquinone anion bands will also be at similar frequencies at 77 and 293 K. We therefore expect to observe TRIR absorption changes at ~ 1495 and ~ 1415 cm^–1^ associated with A_1A_ reduction and recovery at RT. In addition, phylloquinone in the A_1B_ binding site is structurally very similar to that on the A-side, and it is likely that the B-side phyllosemiquinone will also display absorption near 1495 and 1415 cm^–1^ at both 77 K and 293 K.

Both the (P700^+^A_1A_^–^–P700A_1A_) and (P700^+^–P700) FTIR DS in Fig. 2C display peaks around 1482, 1458 and 1431 cm^–1^, so probing absorption changes at these frequencies (in TRIR measurements at RT) will likely result in little or no nanosecond TR absorption changes associated with forward ET from A_1_^–^, but a long-lived (millisecond) contribution due to P700^+^ is expected.

**Fig. 3:**
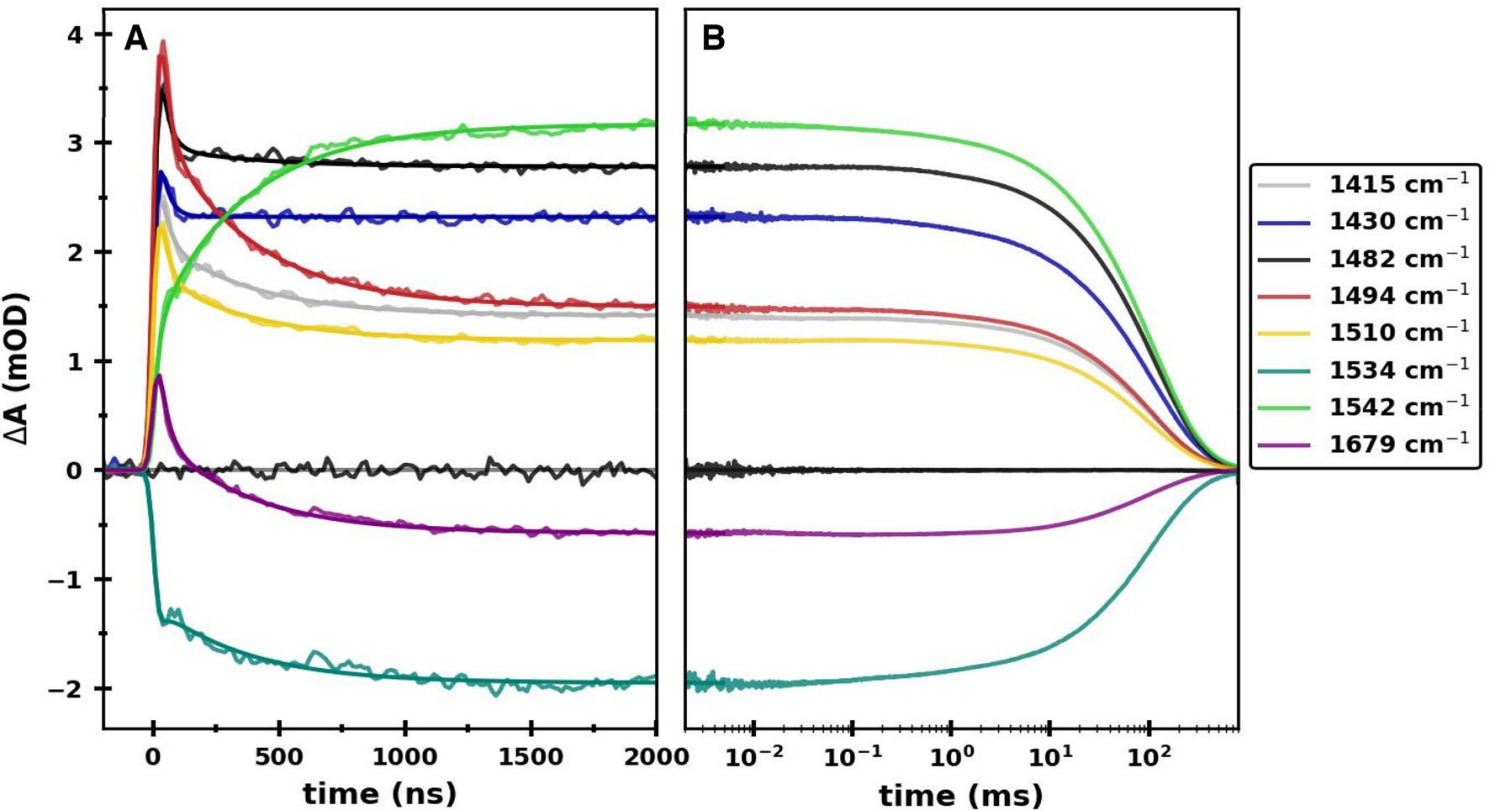
Transient absorption changes at 1415 (*grey*), 1430 (*blue*), 1482 (*black*), 1494 (*red*), 1510 (*yellow*), 1534 (*dark green*), 1542 (*light green*) and 1679 (*purple*) cm^−1^ obtained following 532 nm laser flash excitation of PSI samples at RT. The data around zero (*black*) illustrates the noise level in the experiment and is associated with transient data collected at 1482 cm^−1^, in the absence of laser flash excitation. **A** A linear plot showing the absorption changes up to 2 µs after excitation. **B** A semi-logarithmic plot of the same data showing the absorption changes from 2 µs to 800 ms after excitation. The data at all eight wavelengths, between 0 and 5 µs, is fitted simultaneously to a function consisting of two exponential components and a constant, convolved with the IRF. The time constants but not the pre-exponential amplitudes are constrained to be the same at each wavenumber. The fitted functions are shown in (**A**) and (**B**)

RT flash-induced absorption changes at 1415, 1430, 1482, 1494, 1510, 1534, 1542 and 1679 cm^–1^, on a nanosecond to second timescale, are shown in Fig. 3. The kinetic traces in Fig. 3A are shown on a linear timescale, from 0 to 2000 ns. In Fig. 3B the same kinetic traces are shown on a logarithmic timescale, from 2 × 10^–3^ to 8 × 10^3^ ms. All the transients exhibit nanosecond kinetic phases (Fig. 3A). The absorption changes are relatively constant on microsecond timescales, and decay back to zero on a hundreds of milliseconds timescale (Fig. 3B).

Transient data at all the wavelengths indicated in Fig. 3 were subjected to a global analysis fitting procedure outlined in the materials section and the supplementary information section. The transient kinetics were analyzed in the 0–5 μs time range. From this fitting procedure it is found that the data is best described by a function consisting of two exponential components (and a constant, non-decaying component). The fitted functions are also shown in Fig. 3 and are characterized by lifetimes of 33 and 364 ns (see Table S1 in the supplementary information section). The time constants vary slightly when only a subset of the listed transients are included in the global fitting procedure (*not shown*).

**Fig. 4:**
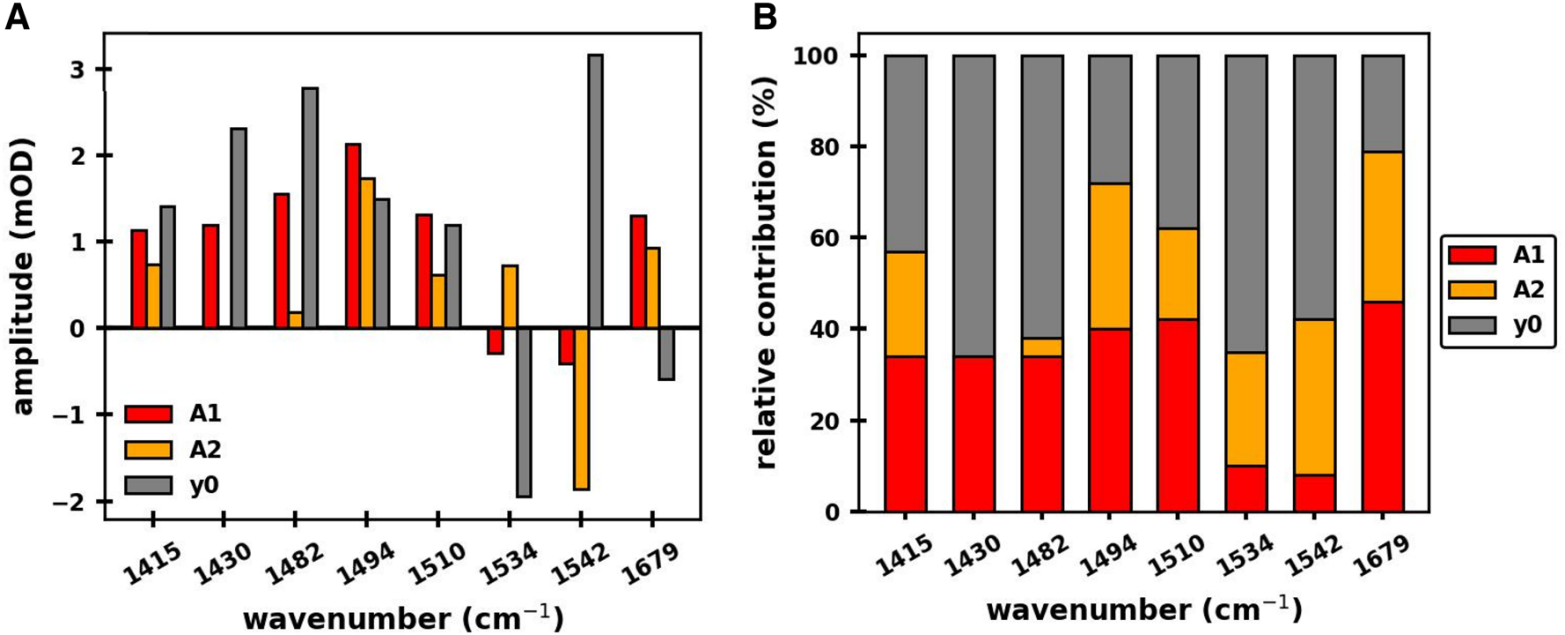
Results from global analysis of the TRIR data in Fig. 3A. **A** Amplitudes, A_1_ and A_2_, of the 33 and 364 ns exponential components, and the amplitude of the non-decaying component (*y*_0_), for each wavenumber. **B** Chart showing the relative contribution of each of the three components for each wavenumber. A_1_, A_2_ and y_0_ relative contributions are proxies for contributions from A_1B_^–^, A_1A_^–^ and P700^+^, respectively

Figure 4A shows the amplitudes of the 33 and 364 ns kinetic phases, along with the amplitudes of the non-decaying phase, for the kinetics at each wavenumber, obtained from the global analysis procedure. The amplitudes are also listed in Table S1. The relative contribution of each of the kinetic phases at each wavenumber is visualized in the bar chart in Fig. 4B.

**Fig. 5:**
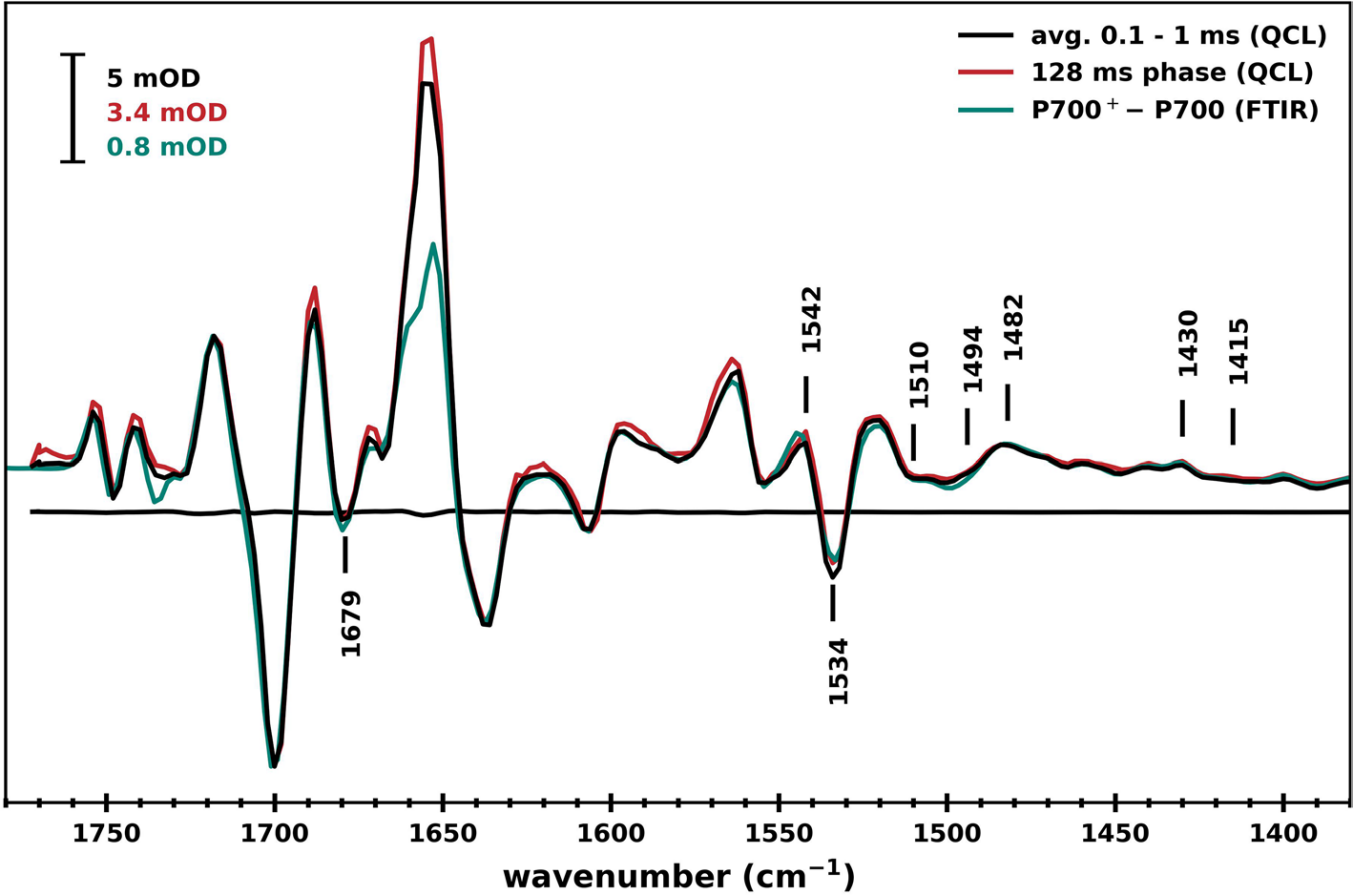
TRIR DS obtained by averaging the flash-induced transients between 0.1 and 1 ms (*black*). Decay associated spectrum of the 128 ms phase obtained by globally fitting TRIR kinetics is also shown (*red*). For comparison a photoaccumulated (P700^+^–P700) FTIR DS obtained for similarly prepared and mounted PSI samples at RT is also shown (*green*). This FTIR DS is also the same as in Fig. 2

Figure 5 shows millisecond TRIR DS extracted from the data collected using the QCL based spectrometer at RT. This data was produced by scanning the QCL output (in 2 cm^–1^ increments) and collecting transients for 6 laser flashes at each wavenumber (300 flashes for wavenumbers below 1550 cm^−1^). In this mode relatively low-sensitivity kinetic data was acquired across the entire 1770–1380 cm^–1^ region. The signal to noise ratio for the data at each wavelength, especially in the ns region, is significantly poorer than that shown in Fig. 3. However, by applying a spectral smoothing algorithm (three neighboring wavenumbers are averaged and assigned to the wavenumber in the middle, resulting in an effective resolution of 6 cm^–1^), and averaging each of the spectra obtained over a given time interval, a high sensitivity TRIR absorption difference spectrum is obtained. Figure 5 (*black curve*) shows the flash-induced TRIR DS resulting from the averaging all 220 spectra collected in the 0.1–1 ms time window, a time range in which absorption changes are minimal (Fig. 3).

We also globally fitted the eight kinetic traces in Fig. 3A, in the 1–800 ms time range, and found that at least five exponential components are needed for a satisfactory fit for the decay back to zero (as judged by the residuals). Time constants extracted are 1.3, 11, 46, 128 and 444 ms, with the 128 ms component being the dominant phase.

Instead of averaging the different spectral blocks derived in this QCL wavelength scanning experiment, we could just directly globally analyze the entire data block (with or without fixed time constants that were derived from fitting the data in Fig. 3B). From this analysis a decay associated spectrum (DAS) associated with the ~ 128 ms phase is obtained, and this DAS is also shown in Fig. 5(*red*). This 128 ms DAS is essentially identical to the average of all the spectra in the 0.1—1 ms range. It is also identical to the average of the spectra obtained in the 1–800 ms range (not shown). Also shown in Fig. 5 is a photoaccumulated (P700^+^–P700) FTIR DS obtained at RT, employing 4 cm^–1^ spectral resolution (*green*) (from Fig. 2). The similarity between the photoaccumulated FTIR DS and the different TRIR DS is obvious, except for the intensity of the positive peak near 1655 cm^–1^, which is more pronounced in the TRIR DS. The origin of this difference in the TRIR and FTIR DS is not clear at present. There are other very small differences in the TRIR and FTIR DS in Fig. 5, for example the absorption change near 1735 cm^–1^, which may relate to the different spectral resolutions in the two experiments.

## Discussion

Nanosecond transient absorption spectroscopy, with 480 nm light, is often used to probe forward ET from A_1_^–^ to F_X_ in PSI at RT (Agalarov and Brettel 2003; Badshah et al. 2018). The positive absorption change at 480 nm is due to an electrochromic effect on a chlorophyll pigment (likely A_0_) caused by the electron residing on the A_1_ pigment (Bautista et al. 2005). The absorption changes at 480 nm were shown to decay biphasically with time constants of 11 and 340 ns at 293 K in PSI from *Synechocystis* sp. PCC 6803 (Agalarov and Brettel 2003). The two time constants have been shown to be consistent with ET down the B– and A–branches in PSI (Guergova-Kuras et al. 2001).

Since absorption changes on the nanosecond timescale are associated with forward ET from A_1_^–^ to F_X_, and changes that do not decay/grow on a nanosecond-microsecond timescale are associated mainly with P700 or P700^+^ (as changes associated with F_X_/F_X_^–^ are minimal in the 1800–1200 cm^–1^ region), we can separate IR absorption contributions from the different pigments based on their time evolution.

For the 1494 cm^–1^ kinetic, the 33 and 364 ns phases have large amplitudes and are well resolved (Figs. 3 and 4A). The 1494 cm^–1^ band in the TRSS FTIR DS at 77 K (Fig. 2C) is due to the C_1_$\dddot -$O group of PhQ^–^ in the A_1A_ binding site (Rohani et al. 2019). For the 1494 cm^–1^ absorption change, 72% of the positive, flash-induced absorption change decays on the nanosecond timescale, with 40 and 32% of the amplitude being associated with the 33 and 364 ns time constants, respectively (Fig. 4B, Table S1). A long-lived positive feature contributing to 28% of the initial absorption change remains following the nanosecond kinetics. As indicated above, the A_1_, A_2_ and y_0_ relative contributions (outlined in Fig. 4B) are proxies for relative contributions from A_1B_^–^, A_1A_^–^ and P700^+^. Therefore, at 1494 cm^–1^, A_1A_^–^, A_1B_^–^ and P700^+^ all have a positive absorption change. This observation also follows from the photoaccumulated and TRSS FTIR DS in Fig. 2C.

The kinetic at 1510 cm^–1^ exhibits similar absorption changes to the one at 1494 cm^–1^ (Fig. 4B), but with a less pronounced 364 ns phase (Fig. 4A). A positive band is also observed at 1511 cm^–1^ in the microsecond TRSS FTIR DS at 77 K, with an intensity that is considerably lower than that at 1495 cm^−1^. The similarity in the relative amplitudes of the nanosecond phases at the two wavelengths at RT, with that in the TRSS FTIR DS at 77 K, suggests that the bands at 1511 and 1495 cm^−1^ at 77 K likely corresponds to bands at similar wavenumber at RT, and that the 33 and 364 ns phases at 1510 cm^–1^ are likely due to A_1B_^–^ and A_1A_^–^, respectively (as is the case for the 1495 cm^−1^ band). The 33 and 364 ns phases in the kinetic at 1510 cm^−1^ at RT are likely associated with quinone ring C$\dddot -$C modes of A_1B_^–^ and A_1A_^–^, respectively. It is well known that semiquinone ring C$\dddot -$C modes have higher frequency vibrations than C$\dddot -$O modes (Bauscher and Mantele 1992; Bauscher et al. 1990).

The absorption changes at 1679 cm^–1^ displays an intense 33 ns phase (46%), with smaller contributions from the 364 ns (33%) and non-decaying (21%) phases. The non-decaying phase of the 1679 cm^–1^ kinetic is weakly negative, as is the amplitude in the photoaccumulated FTIR DS in Fig. 2B, C. So, the 1679 cm^–1^ kinetic indicates that during ET from A_1_^–^ to F_X_, the absorption change is initially positive and the nanosecond bioenergetics lead to a negative absorption change that is due to P700^+^. The species responsible for these nanosecond phases at 1679 cm^−1^ are not clear at present. They could be associated with amide I vibrations from amino acids near A_1_, or with keto carbonyl modes of the A_–1_ or A_0_ pigments (Sivakumar et al. 2005). This is a topic that will be further investigated in the future.

The flash-induced absorption increase at 1482 cm^–1^ occurs within the time resolution of the instrument, followed by a fast 33 ns decay (Fig. 3B, *black*) with little further absorption changes on the hundreds of nanoseconds to microseconds timescale (the 364 ns phase is of very low amplitude (Fig. 4B)). The 33 ns and non-decaying phases account for 34 and 61% of the absorption difference signal at 1482 cm^–1^ (Fig. 4B and Table S1), respectively. However, there is considerable uncertainty in the amplitude for the 33 ns phase. The fact that we observe both positive and negative amplitudes for the 33 ns phase at the different wavelengths (Table S1) confirms that this phase is not an instrumental artefact. However, the 33 ns phase is close to the temporal resolution limits of the instrument (estimated to be 17 ns), and the amplitudes of this phase at the different wavelengths correspondingly have large error (see discussion of this topic in the supplementary information section).

At 1482 cm^–1^, given that there is a 33 ns phase and essentially no 364 ns phase (Table S1), it is unlikely that the 33 ns phase is associated with molecular groups of A_1B_^–^ itself, because then a corresponding 364 ns phase might also be expected near 1482 cm^–1^ associated with A_1A_^–^. Note that we presume that A_1B_^–^ and A_1A_^–^ have absorption bands at similar frequencies, as they do at 1494 and 1415 cm^–1^.

What the 33 ns phase at 1482 cm^–1^ is due to is not clear, possibly an amino acid between A_1B_ and F_X_. Since the 33 ns feature at 1482 cm^–1^ should reflect B-branch and not A-branch ET, we speculate that we have uncovered a spectral observable indicating an asymmetry in the A– and B–branch ET process.

This type of asymmetry between the fast and slow phases may potentially be observed at other wavelengths, such as at 1542 cm^–1^, where the 33 ns phase is of much lower amplitude than the 364 ns phase (Fig. 4B). However, this latter difference may also reflect a branch-utilization asymmetry. In cyanobacterial PSI the A/B branching ratio is roughly 80/20 (Makita and Hastings 2015), respectively. So, the differences in the amplitudes of the two phases at 1542 cm^–1^ may reflect this branch-utilization asymmetry. From the data in Fig. 3A, it is clear that the 33 ns phase is very poorly resolved at 1542 cm^–1^, and also at 1534 cm^–1^, and it is better to refrain from  making assertions based on the amplitude of the 33 ns phase at these frequencies.

In Fig. 3B, following the decay of the nanosecond phases (from ~ 5 μs onwards) a pronounced positive/negative absorption change is observed at 1542/1534 cm^–1^, respectively, that subsequently decays with a time constant of ~ 128 ms. From Table S1 (and Fig. 4A), the non-decaying phase has an amplitude of + 3.17/− 1.95 at 1542/1534 cm^–1^, respectively. In contrast, the TRIR DS in Fig. 5 indicates a higher negative amplitude at 1534 cm^–1^ compared to the corresponding positive amplitude at 1542 cm^–1^. The solution to this issue is to recognize that the broad positive absorption of the P700^+^ state (Fig. 2A, B) also decays in ~ 128 ms. In Fig. 2B one observes that the negative signal at 1534 cm^–1^, relative to the zero line, is considerably less than the positive amplitude of the signal at 1542 cm^–1^ (relative to the zero line), and this amplitude ratio is in line with the millisecond-TRIR kinetic data in Fig. 3B.

Although the absorption difference features in the kinetics in Fig. 3 appear well resolved, the 33 ns kinetic phase is close to the instrumental time resolution. The instrumental time resolution is determined mostly by the response of the IR detector with integrated preamplifier (10 MHz cut-off frequency) and the sampling rate of the A/D converter, where the smallest possible bin size is 15 ns. In our global analysis curve-fitting procedures, the instrument response function (IRF) is well modelled by a Gaussian function with standard width of 17 ns (see discussion in the supplementary information section). Given the 17 ns IRF, and the limited data sampling, the amplitudes of the 33 ns phase at the different wavenumbers is difficult to pinpoint with high precision. Nevertheless, the 33 ns phase is clearly discernable at many wavelengths (Fig. 3A) and is not artefactual. The exact time constant and amplitudes of the 33 ns phase at the different wavelengths are poorly determined, however, depending on the details of the fitting procedure (see the supplementary information section). This is an important consideration. For example, if the 33 and 364 ns phases in the kinetic at 1494 cm^–1^ are due to forward ET from A_1B_^–^ and A_1A_^–^, one might expect the 364 ns phase to exhibit a similar, or up to a two-to-four times larger amplitude, than the 33 ns phase, simply because the branching ratio in PSI is expected to be in the 50/50 to 20/80 range (Redding and van der Est 2006). This is clearly not found to be the case for the 1494 cm^–1^ kinetic data (Fig. 4B). Using an alternate fitting procedure, where zero time for the kinetics is manually adjusted, and the instrument response function is not included in the fitting, the amplitude of the 33 ns phase relative to the 364 ns phase can fall to ~ 40/60 (see discussion in the supplementary information section). So, caution should be exercised at present on building theses based on the amplitude of the 33 ns phase in the various kinetics.

On the other hand, it may be the case that the amplitude of the 33 ns phase is truly larger than the 364 ns at 1494 cm^–1^ (as indicated in Fig. 4B), and if this is the case it could mean that an alternative model of ET needs to be considered, such as the radical pair equilibrium model (Santabarbara et al. 2019). A final answer may have to await the production of kinetic data with improved temporal resolution.

In the analysis of the millisecond-TRIR data in Fig. 3B we found that several exponential components are needed to accurately fit the data on the millisecond timescale. This is in line with previous reports of multi-exponential P700^+^ recombination kinetics that were probed at 820 nm (Vassiliev et al. 1997). In these previous studies DCPIP was used as an electron acceptor, while here we used ascorbate mediated by PMS as an electron donor to P700^+^. In either case the conclusion is that the bioenergetics of radical pair recombination in isolated PSI samples is complex. Attempting to fully disentangle the millisecond bioenergetics is beyond the scope of this manuscript. In spite of the complex millisecond TR bioenergetics, however, Fig. 5 demonstrates that the dominant millisecond DAS (or the average of spectra collected over 0.1–1 ms) agrees well with the photoaccumulated (P700^+^–P700) FTIR DS. The origin of the extra intense positive feature near 1650 cm^–1^ in the TRIR DS is unclear at present. It may be related to intense amide I and water absorption of the samples used in the TRIR experiments.

Millisecond time resolved IR methods (Lorenz-Fonfria 2020; Mezzetti 2020), including rapid-scan FTIR techniques (Mezzetti 2010; Mezzetti et al. 2002), are well-developed and have been widely used for the study of photoactive proteins. Thus, the millisecond time resolved data presented here is not a particularly significant advance. However, Fig. 3 demonstrates the utility of our QCL-based instrument in that it has the capability of simultaneously collecting data on all timescales from nanoseconds to seconds.

## Conclusions

Microsecond TRSS FTIR DS at 77 K for PSI from *T. vestitus* are presented here for the first time. Photoaccumulated (P700^+^–P700) FTIR DS for PSI from *T. vestitus* at both 77 and 293 K, are also presented here for the first time. To greatly extend upon this work PSI from *T. vestitus* was also studied using nanosecond TRIR DS at 296 K. This approach allowed us to probe ET down both branches in PSI. We show that in PSI at 296 K ET down the A– and B–branches is characterized by time constants of 33 and 364 ns, respectively, in good agreement with visible spectroscopy studies.

### Supplementary Information

Below is the link to the electronic supplementary material.

Supplementary file1 (DOCX 581 KB)

## Abbreviations

C=O: Carbonyl
Chl *a*: Chlorophyll *a*
DAS: Decay associated spectrum
DS: Difference spectra/spectrum/spectroscopy
ET: Electron transfer
FTIR: Fourier-transform-infrared
H-bond: Hydrogen bond
IRF: Instrument response function
μs, ms, ns: Micro-, milli-, and nanosecond
NQ: 1,4-Naphthoquinone
PhQ: Phylloquinone (2-methyl-3-phytyl-1,4-naphthoqiuinone)
PSI: Photosystem I
PSII: Photosystem II
*Thermosynechococcus vestitus* BP-1: *T.* *vestitus*
TRIR: Time-resolved infrared
TRSS: Time-resolved step-scan
