# Supplementary material for: Nanosecond time-resolved infrared spectroscopy for the study of electron transfer in photosystem I
Source: Photosynth Res. 2023 Jul 7;159(2-3):229–39. doi: 10.1007/s11120-023-01035-9 (PMC10991071; doi:10.1007/s11120-023-01035-9)
Supplement: Supplementary file 1 — Supplementary file1 (DOCX 581 KB) [file 11120_2023_1035_MOESM1_ESM.docx]

**Supplementary Information**

In support of

**Nanosecond Time-Resolved Infrared Spectroscopy for the Study of Electron Transfer in Photosystem I**

Sarah M. Mäusle^1^, Neva Agarwala^2,3^, Viktor G. Eichmann^1^, Holger Dau^1,*^, Dennis J. Nürnberg^1,4,^* and Gary Hastings^2,^*

**1. Heat artefact correction**

The TRIR DS data is affected by an excitation flash induced heating artefact that decays on the millisecond time scale. The amplitude of the changes associated with the heat artefact grows linearly with excitation intensity.

As indicated in the main manuscript, all TRIR DS data were acquired using saturating laser flashes at low (~0.1 mJ/mm^2^) and high (~0.3 mJ/mm^2^) excitation intensity. When subtracting the transients at low intensity from these at high intensity (Fig. S1, *blue* and *orange* traces), the signals due to PSI photochemistry cancel and all that remains is the absorption changes due to the heating artefact (*green*). This artefactual transient is fit to a sum of exponentials (*black*) and scaled to match the excitation intensity of the low and high intensity transients (*dashed blue* and *orange*). These scaled artefact transients are then subtracted from the original transients, resulting in transients that are corrected for heat artefact contributions (*red*).

**
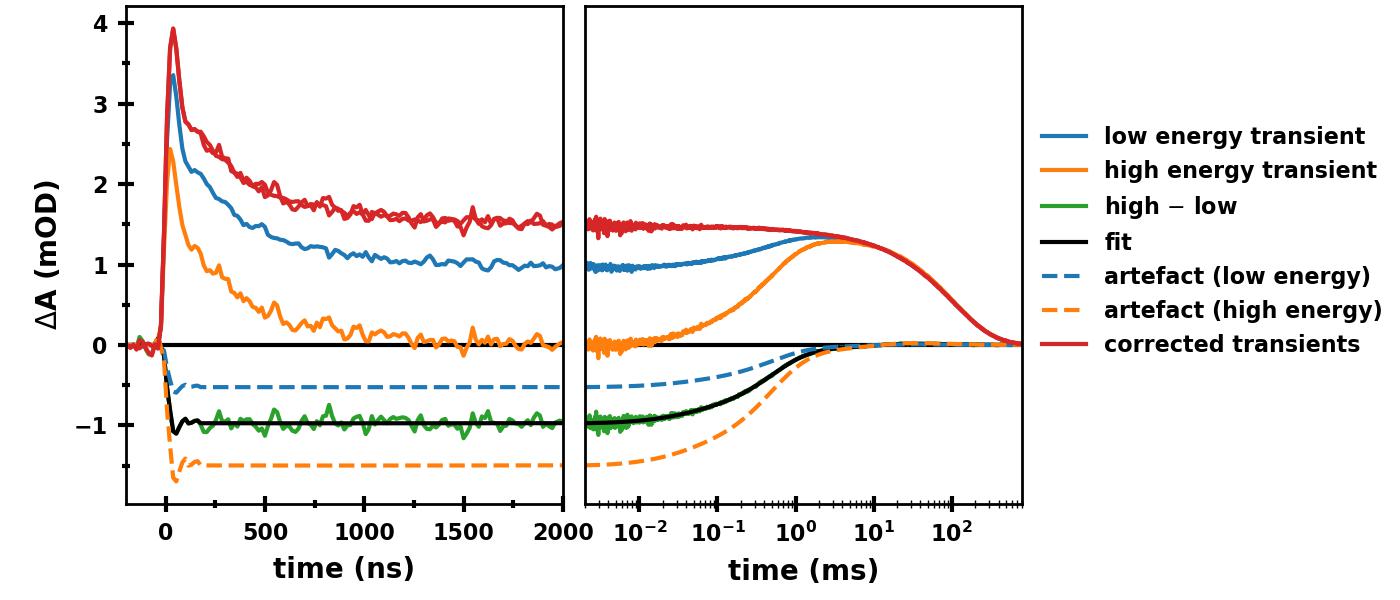
**

**Figure S1: Heat artefact correction.** Transients at 1494 cm^–1^ after excitation with saturating laser flashes of low (*blue*) and three times higher energy (*orange*). The difference between the high and low energy transients, which corresponds to the heat artefact, is also shown (*green*). The heat artefact is fit to a sum of exponentials (*black*) and scaled to match the excitation energy of the low and high energy transients (*dashed blue* and *dashed orange*). Subtracting the scaled artefacts from the low and high energy transients results in heat artefact corrected transients (*red*).

**2. Details on the global analysis fitting procedure and calculated fit parameters**

Table S1 shows the calculated parameters from globally fitting the eight transients shown in Fig. 3 to the following equation: $y\left( t,\nu\right)= y_{o}+A_{1,\nu}e^{-t/\tau_{1}}+ A_{2,\nu}e^{-t/\tau_{2}}$.

As discussed in the main manuscript, the 33 ns component is close to the instrumental temporal resolution. To improve the fit quality, the above equation was iteratively convolved with an instrument response function (IRF) during the least squares minimization procedure. In our analysis The IRF was assumed to be Gaussian:

$\text{IRF}= e^{\frac{{-(t-t_{o})}^{2}}{{2\sigma}^{2}}}$, with σ = 17 ns and *t*_0_ = 0 (Fig. S2, *blue*).

In an ideal experiment, with infinitesimally short IRF, we would expect the absorption difference to be zero until *t* = 0, at which point the maximal signal would be reached instantaneously (Fig. S2, *yellow*). However, we instead observe an absorption increase over a few tens of nanoseconds (*black*). Convolving the fitted exponential curve with the Gaussian IRF (*blue*) results in a fitted curve that simulates well the experimental absorption increase, as well as the subsequent decay (*red*).


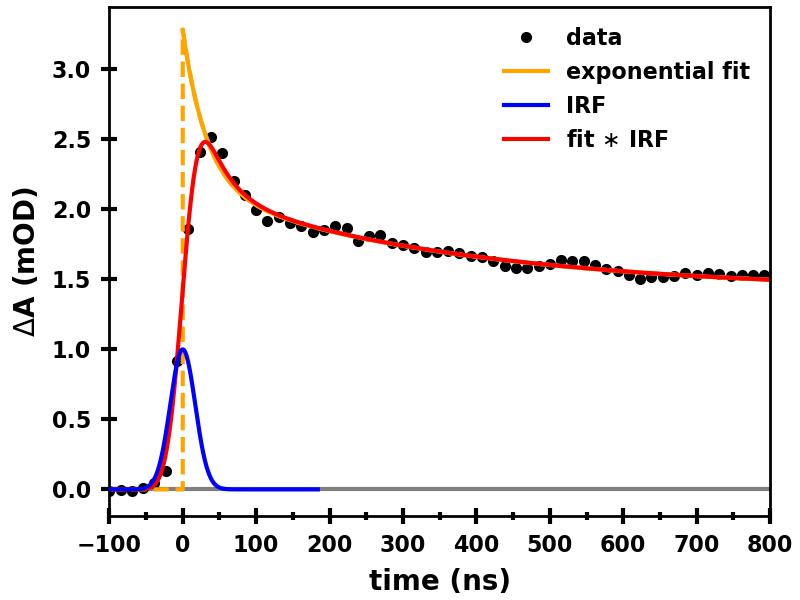


**Figure S2**: Visualization of the used fit approach (with IRF convolution). The difference absorption from -100 to 800 ns at 1415 cm^–1^ is shown in *black*. The sum of two exponentials (with τ_1_ = 33 ns and τ_2_ = 364 ns) plus an offset is shown in *yellow*. A Gaussian curve with σ = 17 ns, representing the IRF, is shown in *blue*. The result of the convolution of the yellow and blue curves is shown in *red*.

**Table S1**: Results from globally fitting the TRIR data in Fig. 3. The percentage that each component contributes to the total absorption change at *t* = 0 is indicated in parenthesis. The given error ranges are the parameter uncertainty ranges calculated from the covariance matrix provided by the software here used in the least squares optimization process (scipy.optimize.least_squares, Python 3.7).

| *ν* (cm^–1^) | A_1_ (mOD)  *τ*_1_ = 33±2 ns | A_2_ (mOD)  *τ*_2_ = 364±5 ns | y_0_ (mOD) |
| --- | --- | --- | --- |
| 1415 | 1.13 ± 0.06 (34%) | 0.74 ± 0.02 (23%) | 1.41 ± 0.01 (43%) |
| 1430 | 1.2 ± 0.12 (34%) | 0.0 ± 0.04 (0%) | 2.32 ± 0.02 (66%) |
| 1482 | 1.56 ± 0.11 (35%) | 0.19 ± 0.03 (4%) | 2.78 ± 0.01 (61%) |
| 1494 | 2.13 ± 0.11 (40%) | 1.74 ± 0.04 (32%) | 1.5 ± 0.01 (28%) |
| 1510 | 1.32 ± 0.06 (42%) | 0.62 ± 0.02 (20%) | 1.19 ± 0.01 (38%) |
| 1534 | -0.28 ± 0.12 (10%) | 0.73 ± 0.04 (24%) | -1.95 ± 0.02 (66%) |
| 1542 | -0.41 ± 0.07 (8%) | -1.86 ± 0.03 (34%) | 3.17 ± 0.01 (58%) |
| 1679 | 1.3 ± 0.07 (46%) | 0.93 ± 0.03 (33%) | -0.58 ± 0.01 (21%) |

**3. Alternative Fitting Approach**

The data was shifted along the x-axis so that t = 0 is where the data would have its initial peak, if it were deconvolved from the IRF (like the yellow curve).

Instead of attempting to account for the instrumentation response, a simpler approach can be undertaken, in which *t* = 0 is determined to be at the initial peak of the transient absorption changes at each wavenumber following laser excitation (Fig. S3C). The data is then globally fit to a sum of exponentials from 0 – 5 µs, but without inclusion of the IRF. This fitting approach results in time constants of 25 and 355 ns. While the time constants calculated are similar to the ones calculated using the IRF, there are larger differences in the calculated amplitudes (compare Fig. S4 to Fig. 4 in the main manuscript). These amplitude differences are especially pronounced for the faster nanosecond phase.

**A**

**C**

**B**


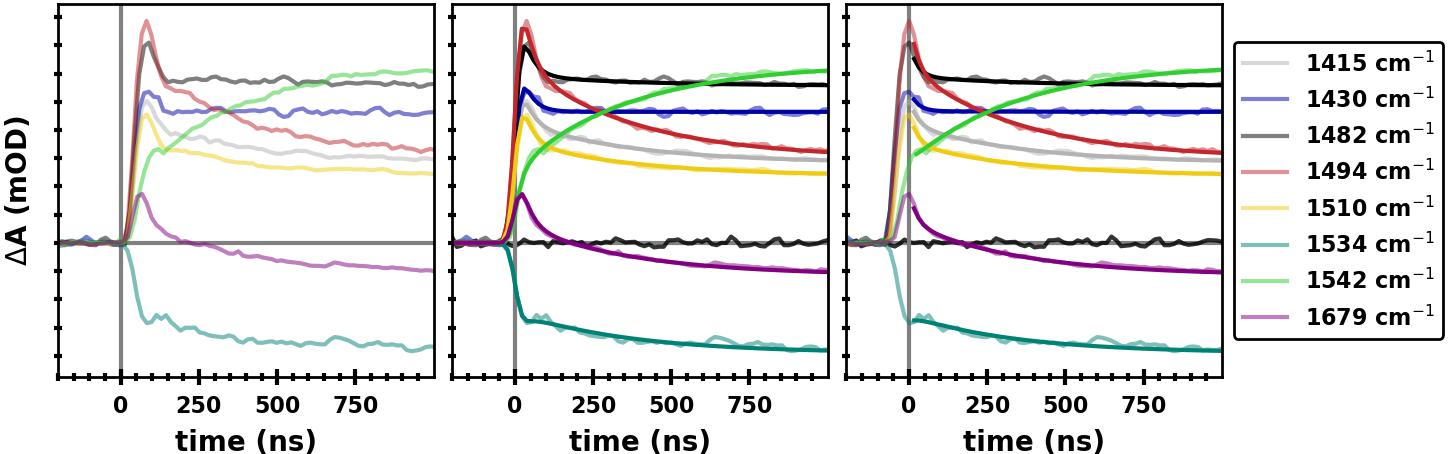


**Figure S3**: Alternative choices of the time axis offset (*t* = 0) for transient absorption changes at 1415 (*grey*), 1430 (*blue*), 1482 (*black*), 1494 (*red*), 1510 (*yellow*), 1534 (*dark green*), 1542 (*light green*) and 1679 (*purple*) cm^–1^. The transients were recorded following 532 nm laser flash excitation of PSI samples at room temperature (same data as shown in Fig. 3 of the main manuscript). (A) Transients with *t = 0* as determined by the photodiode data, taking into account the rise time of the photodiode. (B) Transients with *t* = 0 as used for the fits involving IRF deconvolution (see also Section 2 above). (C) Transients shifted such that *t =* 0 coincides with the initial peak in the transient data. The fits for the main fitting approach and the alternative fitting approach are shown in (B) and (C), respectively.


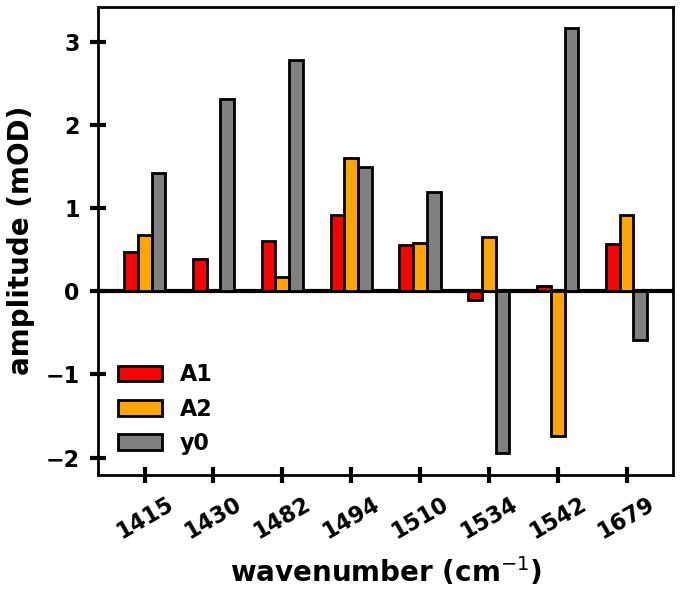


**A**


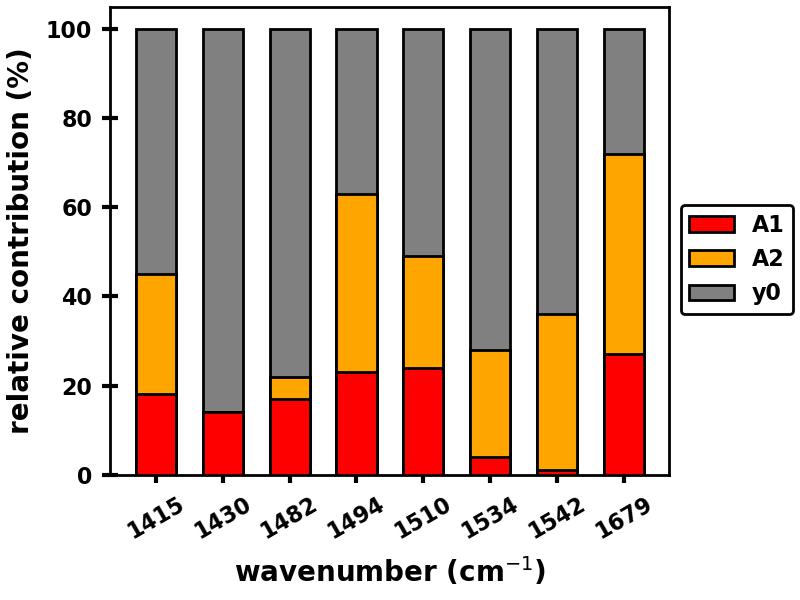


**B**

**Figure S4**: Results from global analysis of the TRIR data in Fig. S3C (where the peak of the transients were shifted to be at *t* = 0). (A) Amplitudes, A_1_ and A_2_, of the 25 and 355 ns exponential components (derived from the fitting procedure), and the amplitude of the non-decaying component (*y*_0_), for each wavenumber. (B) Chart showing the relative contribution of each of the three components for each wavenumber. A_1_, A_2_ and y_0_ relative contributions are proxies for contributions from A_1B_^–^, A_1A_^–^ and P700^+^, respectively. Comparison to the fit parameters shown in Fig. 4 of the main manuscript illustrates the influence of using the refined fit approach using IRF convolution (Fig. 4) and a more approximative approach (Fig. S3C and S4).

**4. Additional Data**

As discussed in the main manuscript, P700^+^ displays very broad and intense absorption bands throughout the 4000-1200 cm^–1^ region. This broad electronic absorption overlies the vibrational difference bands of the cofactors, leading to an upshift of the difference bands of the cofactors. Above ca. 1760 cm^–1^, there are no cofactor vibrational bands, but because of the broad electronic absorption of P700^+^ the transient signals are not zero. Fig. S5 (*red*) shows the flash-induced transient signal at 1770 cm^–1^, which shows a positive difference signal. This signal is constant throughout the entire nano- and microsecond time range, decaying back to zero on the millisecond time scale. The transient signal at 1510 cm^–1^ is compared to the 1760 cm^–1^ signal in Fig. S5. The millisecond decay kinetics are identical at both wavenumbers (Fig. S5B). Indeed, if the other transients shown in Fig. 3 of the main manuscript are normalized in the millisecond time range, they all show the same behavior (*not shown*). This observation further supports the conclusion that the broad difference absorption is associated with P700^+^.

**B**

**A**

*
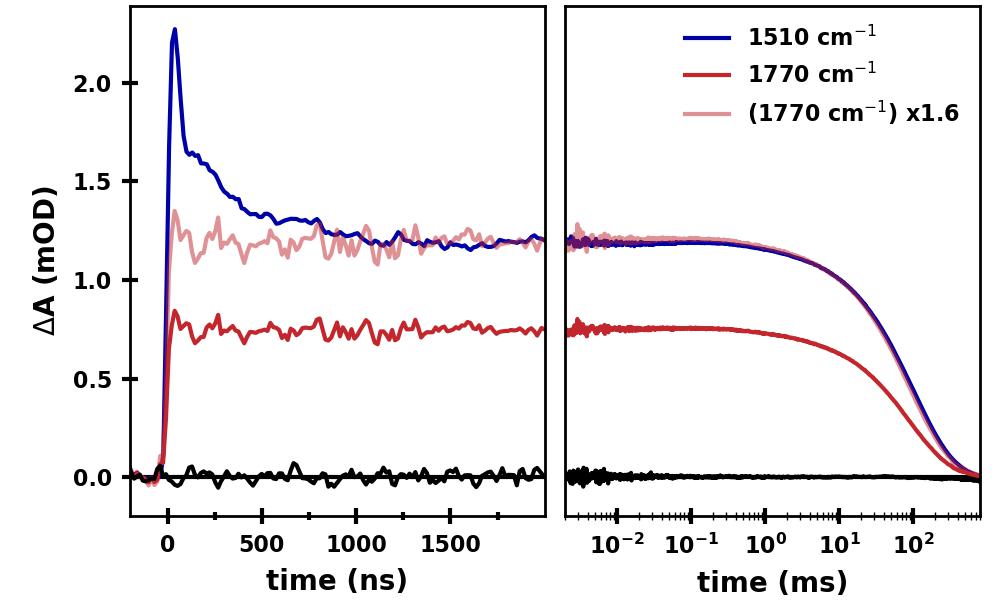
*

**Figure S5**: Transient absorption changes at 1510 (*blue*) and 1770 cm^–1^ (*red*). (A) A linear plot showing the absorption changes up to 2 µs after excitation. (B) A semi-logarithmic plot of the same data showing the absorption changes from 2 µs to 800 ms after excitation. The data at 1770 cm^–1^ multiplied with a factor of 1.6 (*pink*) is also shown for better visual comparison with the data at 1510 cm^–1^. Transient data at 1770 cm^–1^ collected in the absence of laser flash excitation (*black*) indicates the noise level.
